# Humanised Mice and Immunodeficient Mice (NSG) Are Equally Sensitive for Prediction of Stem Cell Malignancy in the Teratoma Assay

**DOI:** 10.3390/ijms23094680

**Published:** 2022-04-23

**Authors:** Monika Bialecka, Joaquin Montilla-Rojo, Bernard A. J. Roelen, Ad J. Gillis, Leendert H. J. Looijenga, Daniela. C. F. Salvatori

**Affiliations:** 1 Anatomy and Physiology, Department Clinical Sciences, Faculty of Veterinary Medicine, Utrecht University, Yalelaan 1, 3584 CL Utrecht, The Netherlands; m.bialecka-dejong@uu.nl (M.B.); j.montillarojo@uu.nl (J.M.-R.); b.a.j.roelen@uu.nl (B.A.J.R.); 2 Princess Máxima Center for Pediatric Oncology, Heidelberglaan 25, 3584 CS Utrecht, The Netherlands; a.j.m.gillis@prinsesmaximacentrum.nl (A.J.G.); l.looijenga@prinsesmaximacentrum.nl (L.H.J.L.)

**Keywords:** hPSCs, hiPSCs, teratoma assay, pluripotency, malignancy, humanised mice

## Abstract

The use of human pluripotent stem cells (hPSCs) in regenerative medicine has great potential. However, it is important to exclude that these cells can undergo malignant transformation, which could lead to the development of malignant tumours. This property of hPSCs is currently being tested using the teratoma assay, through which cells are injected into immunodeficient mice. Transplantation of stem cells in immunocompromised recipient animals certainly has a much higher incidence of tumour formation. On the other hand, the results obtained in immunodeficient mice could indicate a risk of tumour formation that is practically not present in the human immunocompetent recipient. The presence of a humanised immune system might be more representative of the human situation; therefore, we investigated if the demonstrated malignant features of chosen and well-characterised stem cell lines could be retrieved and if new features could arise in a humanised mouse model. Hu-CD34NSG^TM^ (HIS) mice were compared side by side with immunocompromised mice (NSG) after injection of a set of benign (LU07) and malignant (LU07+dox and 2102Ep) cell lines. Analysis of the tumour development, histological composition, pathology evaluation, and malignancy-associated miRNA expression levels, both in tumour and plasma samples, revealed no differences among mouse groups. This indicates that the HIS mouse model is comparable to, but not more sensitive than, the NSG immunodeficient model for studying the malignancy of stem cells. Since in vivo teratoma assay is cumbersome, in vitro methods for the detection of malignancy are urgently needed.

## 1. Introduction

Human pluripotent stem cells (hPSCs) are defined by their capacity to self-renew and to differentiate towards derivatives of the three germ layers: ectoderm, mesoderm, and endoderm. As such, hPSCs offer great promise in personalised regenerative medicine, according to which patients’ somatic cells can be reprogrammed in vitro and used to study specific drug responses or disease mechanisms. Furthermore, after correction and differentiation, they could potentially be used in autologous transplantation to improve organ efficiency or substitute damaged tissues [1]. However, before prospective use in the clinic, all hPSCs need to be examined for potential malignant potential.

While pluripotency and developmental potential can be tested in silico with transcriptome analysis, by using tools such as PluriTest [2] or in vitro in short term differentiation experiments (in monolayer culture or embryoid bodies), combined with bioinformatic analysis such as Scorecard [3,4], malignancy can be assessed in vivo only by injection of cells into immunodeficient animals (teratoma assay) [5]. To this end, even though animal-based and, despite several calls being made [2,6], still not a standardised test, the teratoma assay is used to assess developmental potential in vivo and identify cell lines with potential malignant properties.

In the teratoma assay, a cell suspension in a culture medium or mixed with extracellular matrix components is injected into, for instance, the testis or under the skin of mice. If the injected cells indeed were pluripotent, the tumours that develop in mice over several weeks contain highly heterogeneous derivatives of three germ layers of different maturity and are called teratomas [7]. 

Histopathologically teratomas derived from stem cells are similar to human germ cell tumours (hGCTs), which originate from altered human germ cells (hGCs). These tumours can additionally contain embryonal carcinoma (EC), yolk sac (YS), and immature neural elements, which are associated with the malignancy of hGCTs [8]. In teratomas derived from stem cells, malignant elements such as EC- and YS-like structures have been histologically described, and they have been mostly observed in teratomas, also containing tissue derivatives of all three germ layers within the same tumour. Additionally, the presence of undifferentiated stem cells has been described in teratomas, although the biological significance of undifferentiated/immature elements in teratomas remains unknown [9,10]. Nevertheless, the presence of these elements is a cause of concern and possibly indicates the malignant potential of a stem cell line. Malignant hGCTs can be diagnosed from liquid biopsies (plasma samples) by the presence of certain miRNAs such as miR371 and miR373 families [11,12]. We have previously shown that potentially malignant elements contained in teratomas generated from cell lines such as hEC cell line 2102Ep and LU07+dox hPSC can also be detected by the same miRNAs that are indicative of malignancy for hGCT in the blood of mice injected with those cells [13]. Despite the existence of the aforementioned in vitro assays used for testing pluripotency, a human-based in vitro model able to detect malignancy of stem cells has not yet been developed.

The teratoma assay is usually performed in various strains of immunodeficient mice. Frequently used NSG mice (NOD.Cg-*Prkdc^scid^ Il2rg^tm1Wjl^*/SzJ) carry mutations that render the immune system deficient in mature B and T cells (*Prkdc^scid^*), macrophages, and dendritic cells, and additionally exhibit disturbed cytokine signalling via IL2 receptors (*IL2rg^null^*), leading to functionally defective NK cells [14]. The results on tumour development after injection of stem cells need to be interpreted in view of the used animal model. The limitations of translating discoveries in rodents into clinical applications are heavily under debate, also in cancer research [15]. Transplantation of stem cells in immunocompromised recipient animals has certainly a much higher incidence of tumour formation [16]. On the other hand, the results obtained in immunodeficient mice could indicate a risk of tumour formation that is practically not present in the human immunocompetent recipient. We reasoned that the presence of a humanised immune system might be more representative of the human situation. Therefore, we investigated if the demonstrated malignant features of chosen stem cell lines could be retrieved and if new features could arise in a humanised mouse model.

Humanised mice have been generated by engraftment of CD34+ human hematopoietic stem cells (hu-CD34+ HSCs) into female newborn NSG mice [17]. In contrast to NSG mice, humanised mice (HIS) have functional CD4+ and CD8+ T cells, macrophages, dendritic, and NK cells derived from these hu-CD34+ HSCs. Despite being used in cancer studies, humanised mice have not yet been evaluated as models for teratoma formation.

Here, we compared the outcome of the teratoma assay in NSG and HIS mice using normal hiPSCs (LU07), hiPSCs with reactivated (doxycycline-inducible) reprogramming factors (LU07+dox), and human embryonal carcinoma cells (2102Ep).

The results indicate that the growth rate of the tumours, as well as histological and pathological features and expression levels of specific-circulating and tumour miRNAs associated with malignancy, do not differ between NSG immunodeficient mice (NSG) and HIS mice with a reconstituted human immune system.

## 2. Results

### 2.1. The Time of Tumour Development Is Similar between Animal Groups 

**Figure 1:**
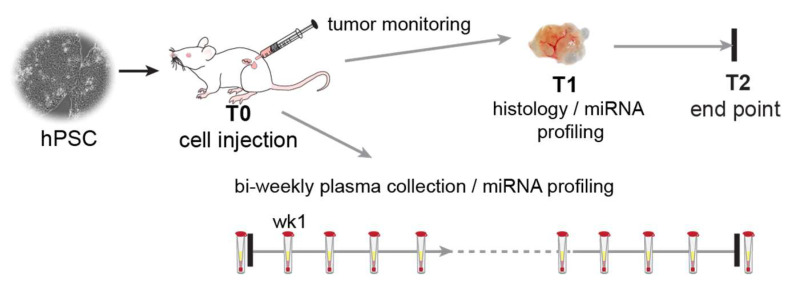
Experimental setup. Schematic overview of the experimental timeline. Each mouse (NSG and HIS) (*n* = 27) was injected with 10^6^ cells in Matrigel and culture medium (T0) and was weekly monitored for tumour growth. Tumour size was measured using a digital calliper. When the tumour reached ~2 cm^3^, it was surgically removed (T1), and the animal was monitored for possible further tumour development till the end of the experiment (T2) when the animals were sacrificed. Plasma samples were collected before cell injection, one week after injection, and subsequently bi-weekly.

We compared tumour formation from known benign hiPSCs (LU07), malignant hiPCSs (LU07+dox), and embryonal carcinoma cells (2102Ep) in the teratoma assay, following a well-established protocol [18]. Each cell line was injected into the flanks of three mice per cell line to generate tumours (Figure 1). The teratoma assay is commonly performed with male NSG mice, while HIS mice are delivered as females. Therefore, we compared the tumour formation in three groups of animals: NSG males, NSG females, and HIS females (total *n* = 27) (Figure S1). We monitored all animals for tumour growth and collected plasma samples for miRNA analysis (Figure 1).

**Figure 2:**
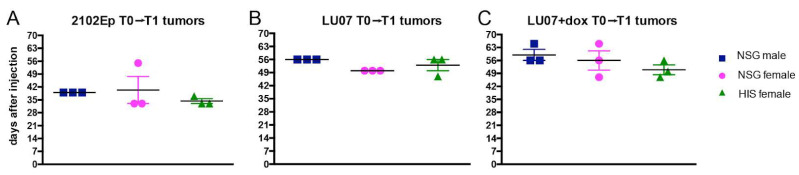
Primary (T1) tumour growth time (from cell injection to first time point of detection) in NSG male, NSG female, and HIS female mice injected with (**A**) 2102Ep, (**B**) LU07, and (**C**) LU07+dox cells. The time was measured from the day of injection of cells to the day when tumours were surgically removed. Number of mice per cell line *n* = 3. Black horizontal bars indicate mean, and error bars indicate standard error of the mean. Statistical analysis was performed using the 2-way ANOVA test. There were no significant differences between NSG males, NSG females, and HIS females.

After cell injection, the mice were analysed for tumour growth; when a tumour reached ~2 cm^3^, it was surgically removed (T1). Tumours derived from 2102Ep cells grew on average for 39 days in NSG males and 40 and 35 days in NSG females and in HIS females, respectively, before surgical removal (T1) (Figure 2A). In the case of LU07-derived tumours, the times of growth before the removal were 56, 50, and 53 days after cell injection for NSG males and females and HIS females, respectively (Figure 2B). Tumours generated from LU07+dox cells (Figure 2B) were removed after 59, 56, and 51 days on average in NSG males, NSG females, and HIS females, respectively (Figure 2C). There were no significant differences in tumour growth (time from cell injection to removal of the tumour, T1) between all animal groups (Figure 2). 

We also assessed the time the tumours were first detectable after cell injection. The tumours that arose from 2102Ep ECs were first detected on day 22 in NSG males, and on day 21 in NSG and HIS females, after injection of stem cells (Figure S2). Tumours generated from LU07 hiPSCs were on average first detected on day 34 in NSG males, and days 27 and 34 in NSG and HIS females, respectively, while tumours from LU07+dox hiPSCs were first palpable by days 41, 27, and 34 in NSG males, NSG, and HIS females, respectively. There were no significant differences in the interval between injection of cells and the first detection of the tumour among NSG male, NSG female, and HIS female mice, per cell line (Figure S2).

### 2.2. LU07 hiPS Form Benign Teratomas, LU07+dox, and 2102Ep Form Malignant Tumours

For all animals, the tumours were allowed to develop until they reached ~2 cm^3^ before they were surgically removed (T1, Figure 1). The experiments were continued for several weeks to monitor if any of the animals developed tumours after T1 was removed. 

Mice injected with the 2102Ep cell line (*n* = 9/9) developed T1 tumours and smaller tumours at the end of the experiment (T2; 8/9) (Figure S3A). Histological analysis revealed typical morphology that is characteristic of EC in both T1 and T2 tumours. All tumours were composed of cells expressing OCT4 and CD30, confirming that these were indeed EC (Figure S3B,C).

**Figure 3:**
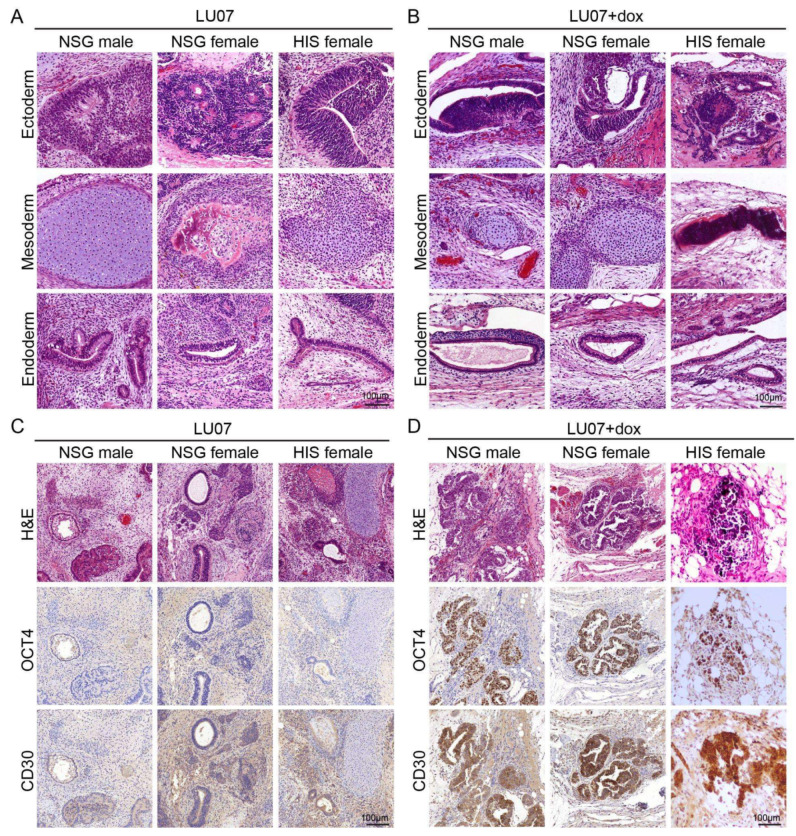
Histopathology analysis of tumours derived from LU07 and LU07+dox in NSG and HIS mice: (**A**,**B**) representative H&E-stained sections showing derivatives of the three germ layers in LU07 and LU07+dox hiPSC, respectively; (**C**,**D**) representative images of OCT4- and CD30-immunostained (brown) areas in LU07 and LU07+dox matched with H&E, showing morphology and marker staining characteristic for EC components. Note that LU07 tumours lack EC components.

All NSG males, NSG, and HIS females injected with LU07 hiPSCs formed T1 tumours. These were teratomas with derivatives from all the three germ layers, where ectoderm was often represented by neural tubes, mesoderm by various stages of cartilage development, and endoderm by different types of epithelium (Figure 3A). Unlike 2102Ep cells, mice injected with LU07 did not give rise to new tumours after T1 removal (Figure S3A). 

LU07+dox hiPSCs also generated tumours containing derivatives of the three germ layers (Figure 3B) but additionally contained areas with EC components characterised by the expression of OCT4 and CD30 (Figure 3D). The presence of EC components was consistent in all NSG males, NSG, and HIS females. We examined sections of the lymph nodes (subiliac and mesenteric, liver, lungs) by H&E staining and found no signs of metastasis in any of the organs (data not shown).

Teratomas tend to be cystic tumours, in which cysts containing fluid are in between solid areas (Figure S4). Possibly, the cystic structures hamper the determination of the actual tumour mass. Therefore, the solid surface areas in tumours generated by LU07 and LU07+dox in NSG male and females and in HIS female mice were determined in serial sections (1:10) from all available slides stained with H&E. The total volume of the solid components of the LU07 and LU07+dox tumours (excluding empty spaces generated by cysts) did not differ significantly between NSG and HIS females (Figure S4).

### 2.3. MiR371a-3p and miR373-3p Indicate the Presence of the Malignant EC Components in Tumours of Both NSG and HIS Mice

We previously [13] showed that after the injection of malignant hiPSCs in mice, high levels of miR-371a-3p and miR-373-3p both in mouse plasma and tumour tissue samples are correlated with the development of tumour-containing EC elements. To determine whether the presence of a humanised immune system in the mouse would affect the efficiency of these miRNAs as predictive markers of malignancy, we analysed bi-weekly and endpoint plasma and tumour samples. 

**Figure 4:**
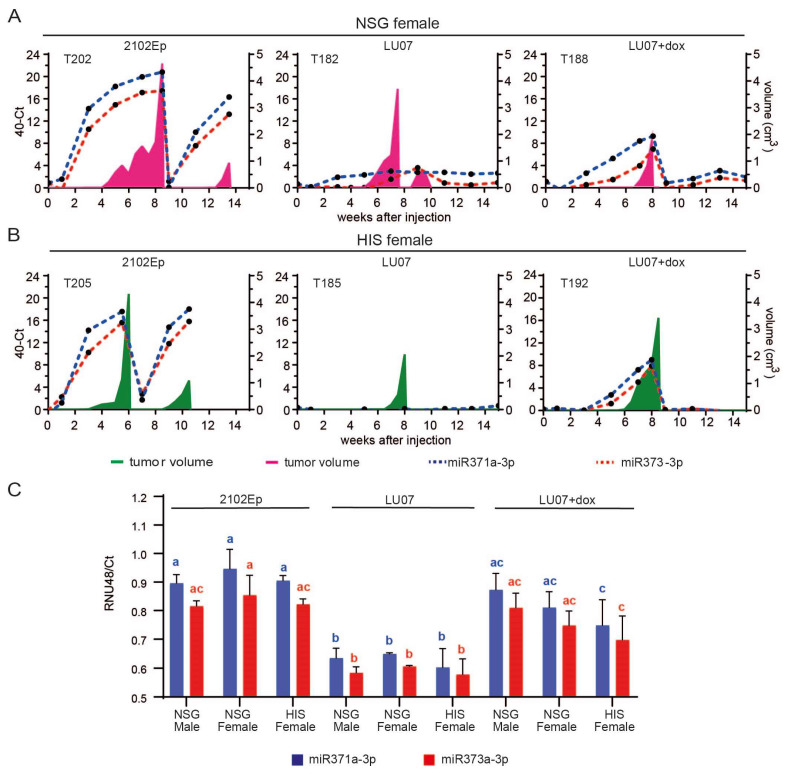
MiR-371a-3p and miR-373-3p expression profile in mouse plasma and tumour samples. Relative levels (40-CT) of circulating miR-371a-3p (blue dotted line) and miR-373-3p (red dotted line) in the plasma of a representative NSG (**A**) and HIS (**B**) mouse xenografted with 2102Ep, LU07, and LU07+dox cell lines. Plasma samples were collected once every two weeks until the end of the experiment (max week 15 from cell injection). Tumour volume is represented in pink for the NSG female and green for the HIS female mice; (**C**) relative levels (RNU48/Ct) of miR-371a-3p and miR-373-3p in the T1 tumour samples of mice xenografted with 2102Ep, LU07 and LU07+dox cells retrieved from tumour sections. Same coloured bars with different letters on top are significantly different (*p* < 0.05). Statistical analysis was performed using the 2-way ANOVA test. Error bars indicate standard deviations of three biological replicates.

The accumulation of circulating miRNAs in plasma correlated with tumour growth in NSG females injected with 2102Ep and LU07+dox, both for T1 and T2 tumours (2102Ep only). Furthermore, the build-up of these miRNAs preceded the first detection of the tumour (Figure 4A, Figure S5A). 

Similar expression patterns were observed for HIS mice, where a high expression of miR-371a-3p and miR-373-3p also preceded the development of tumour-containing EC components (LU07+dox and 2102Ep). In addition, these miRNAs were not detected for HIS mice injected with LU07 (Figure 4B, Figure S5B). NSG males injected with 2102Ep showed a similar pattern of accumulation of circulating miR-371a-3p and miR-373-3p preceding tumour development (Figure S5C), in line with our previous report [13]. Once the tumours were removed (T1), the levels of miRNAs were undetectable in LU07- and LU07+dox-injected mice. In T1 tumour samples, higher expression of miR-371a-3p and miR-373-3p also indicated the malignant nature of the injected cell line, as levels of both miRNAs in mice injected with 2102Ep and LU07+dox were significantly higher than those in LU07-derived xenografts. Nonetheless, no significant differences were found in miRNA levels among mouse groups injected with LU07, LU07+dox, or 2102Ep (Figure 4C).

A single LU07 tumour in one HIS female (T186) presented increased levels of both miRNAs in plasma samples and tumour samples even though histological analysis did not reveal any EC component. This particular tumour had rare, small pockets of OCT4-expressing cells, which did not have EC morphology and did not express CD30 (data not shown), and therefore, were classified as undifferentiated cells.

### 2.4. Macrophages Are Present in the Fibrous Tissue Surrounding the Tumour but Rarely in the Tumour Parenchyma

**Figure 5:**
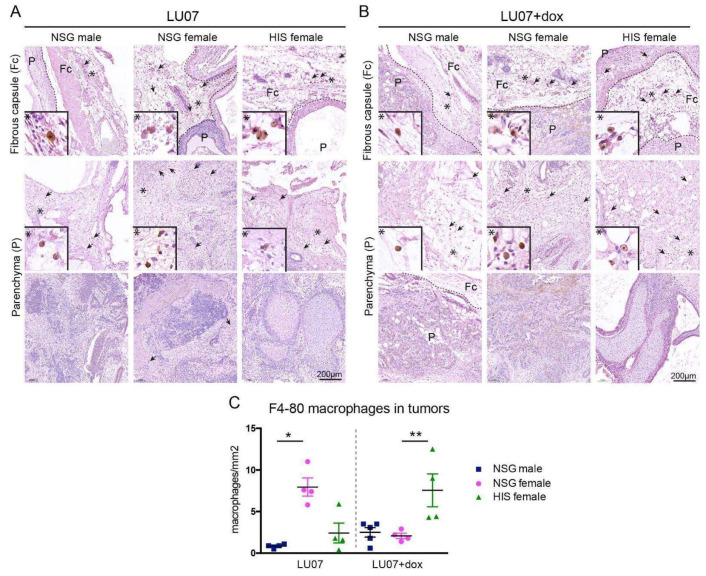
Presence of macrophages in tumours derived from stem cells in HIS and NSG: (**A**) presence of F4-80 stained macrophages in the fibrous capsule (Fc) (top panel) and tumour parenchyma (P, middle and bottom panels) in LU07, and (**B**) LU07+dox derived tumours in NSG males, NSG females, and HIS females. Arrows indicate F4-80 stained macrophages. Dotted line indicates the border of tumour and mouse tissue; asterisks indicate enlarged areas presented in the bottom left of panels; (**C**) calculation of the F4-80 stained macrophage density in tumour parenchyma per mm^2^ of 4–5 tumour sections. * indicates *p* < 0.05; ** *p* < 0.005. Statistical analysis was performed using the Mann–Whitney test.

Since HIS mice contain functional human immune cells, we examined the presence of F4-80 positive macrophages in the hPSC-derived tumours. In all animals, macrophages were present in the tissue surrounding the tumour (fibrous capsule) in varying amounts (Figure 5A,B top panels), independent of the cell line or mouse model. Therefore, the presence of macrophages in tumour parenchyma was examined. No F4-80 positive macrophages were detected in the parenchyma of 2102Ep-derived tumours (data not shown). In tumours generated in NSG males from LU07 hiPSCs, macrophages were sparsely present in the solid part (non-cystic part) of the tumours. Similar results were obtained in NSG females and HIS females, with few F4-80 positive cells in areas of the tumours. In LU07+dox-generated tumours, macrophages were rarely detectable in compact tumour areas. We quantified the number of F4-80 positive cells per mm^2^ of the tissue in serial sections of the tumour sections and indeed HIS females presented significantly higher cell numbers in LU07+dox tumours when compared with NSG males and females (Figure 5C).

## 3. Discussion

Humanised mouse models have been used in various areas of immunology, including allergy, autoimmunity, infectious disease, and cancer [19] but, to our knowledge, not to evaluate the malignancy of stem cells in the in vivo teratoma assay. At the actual advancement of assay development, the teratoma assay remains relevant since it is the only assay to provide an assessment of pluripotency and malignant potential, which are both relevant to the preclinical safety assessment of hPSCs [5].

In a side-by-side comparison, we analysed several hPSC lines with distinct differentiation capacities in the teratoma assay performed in NSG and HIS mice. We compared the time of first appearance and the dynamics of tumour growth generated from benign (LU07) and malignant (LU07+dox and 2102Ep) cell lines and observed no significant differences between NSG and HIS mice. Furthermore, the variations between the cell lines we observed reflected the nature of the cell line (fast-growing 2102Ep vs. slow-growing LU07 and LU07+dox) and were similar in NSG and HIS mice. Histopathological features were similar in HIS and NSG mice and were consistent with our previous data [13]. LU07 hiPSCs all formed teratomas containing the three germ layers and were lacking undifferentiated areas, as shown by H&E and OCT4 staining. By contrast, LU07+dox cells, previously reported to be malignant [13,18], generated teratomas composed of tissues derived from three germ layers but with the presence of EC components, which closely resembled hECs generated from 2102Ep cells, expressing both CD30 and OCT4 pluripotency markers.

Minimally invasive, liquid biopsies are already used for the diagnosis of hGCTS [20,21]. Using this approach, the cell lines used here (LU07, LU07+dox, 2102Ep) were previously miRNA-profiled after injection into NSG mice [13]. Here, we used the same strategy, by generating tumours using NSG mice as a control to determine whether HIS mice were sensitive enough for malignant miRNA detection. We examined mouse plasma miRNA levels after injection of hPSC at the endpoint of the xenograft and compared these levels with those before injection. We analysed miRNA371a-3p and miRNA373-3p expressions, previously reported to be good predictive markers for malignancy in hGCTs [12,22] and hPSCs [13]. The expressions of these miRNAs in 2102Ep tumours increased before the tumours were visible and decreased after surgical tumour removal. In malignant LU07+dox-injected animals, increased levels of miRNA371a-3p and miRNA373-3p were observed in NSG and HIS mice, which all contained EC components. We confirmed this finding also in HIS mice: miRNAs patterns reflect the histological constitution of the tumours even before tumours are measurable by calliper [13]. We also showed that, after surgical removal of tumours (T1 in HIS and NSG mice), miRNAs are cleared from the circulation, as has also been observed in patients after surgical removal of clinically manifested hGCTs [22,23]. The patterns found in the mouse xenograft models mimic those in hGCT patients. Interestingly, the miR-371 family has been found to be an alternative mechanism for the inactivation of the P53 pathway in hGCTs [24]. TP53 mutations have already been reported in human embryonic stem cells, suggesting that these mutations confer a selective advantage. After sequencing the protein-coding genes (exomes) of 140 independent hESC lines, Merkle et al. concluded that acquisition and expansion of cancer-associated mutations in hPSCs may go unnoticed during most applications and advised careful genetic characterisation of hPSCs and their differentiated derivatives prior to clinical use [25].

Combined, the data presented here suggest that the existence of a partially reconstituted human immune system is not advantageous in a teratoma assay over NSG mice for detecting malignancy of stem cells. In clinical situations, it is more likely that the immune system will be, at least partially, active. Macrophages as a part of the innate immune system are the first line of defence for the organism against pathogens and invading foreign or transformed cells. Generally, M1 macrophages (classically activated macrophages) are responsible for inflammatory response [26,27], while M2 macrophages (alternatively activated macrophages) for parasite infection, tissue remodelling and angiogenesis [28,29]. Once associated with a tumour tissue, tumour-associated macrophages (TAMs) can affect tumour progression and either have tumour-promoting effects [30] or exhibit antineoplastic activity [31]. Therefore, functional macrophages could influence the outcome of the assay. However, the assessment of macrophage infiltration in the tumour in NSG and HIS mice did not reveal major differences between animals. HIS females injected with malignant LU07+dox cells exhibited increased numbers of macrophages, compared with those injected with benign LU07 cells, which might be a consequence of the presence of human macrophages. Alternatively, since TAMs can have the properties of either M1 or M2 macrophages, tumours in HIS mice underwent more tissue remodelling, thereby attracting more macrophages. Interestingly, there was a significant difference between the numbers of F4-80-positive macrophages in NSG males and females injected with LU07. However, since the macrophages in NSG mice are defective [17], this would not affect the immune response.

In conclusion, when a teratoma assay is performed to detect malignancy of a cell line, NSG mice appear equally sensitive as HIS mice.

The teratoma assay raises both ethical and methodological questions. It is performed on mice in which tumours of considerable size need to develop, creating obvious animal welfare issues. Moreover, it is a time-consuming assay that requires expert pathological assessment, which is difficult to quantify and impossible to apply as a routine and large-scale screening tool [32]. Importantly, despite the several calls [6,33], the teratoma assay has never been standardised. Additionally, the generation of HIS mice requires irradiation of newborn animals, adding to animal discomfort [17].

Future research is urgently needed to replace the teratoma assay with in vitro methods based on genetic and epigenetic biomarkers able to identify cell lines with malignant potential.

## 4. Materials and Methods

### 4.1. Cell Lines

Human embryonal carcinoma 2102Ep [34,35] and human-induced pluripotent cells LUMC007iCTRL01 (LU07) were used in this study. The generation of the LU07 cell line with the doxycycline-inducible TetO–FUW–OSKM construct was described previously [18]. Activation of the transgenes results in continuous expression of reprogramming factors (OCT3/4, SOX2, KLF4, MYC), rendering the cell line differentiation defective. LU07 cells were cultured on VitronectinXF (Stem Cell Technologies)-coated, standard tissue culture plates in mTeSR-E8 (Stem Cell Technologies) culture medium, refreshed daily, and passaged weekly as clumps with a Gentle Cell Dissociation Reagent (GCDR, Stem Cell Technologies); 2102Ep cells were cultured as described [18]. The LU07 cell lines without activation of the transgenes are abbreviated in this manuscript as LU07. Expression of the transgenes was activated and maintained by the addition of doxycycline (2 µg/mL final concentration) in culture media for 3 days prior to use for injections into animals. These cells are abbreviated as LU07+dox [18]. 

### 4.2. FACS Analysis 

On the day of cell injection, LU07 and LU07+dox cells were tested for pluripotency. Single-cell suspensions were processed with Fix&Perm Cell fixation and Permeabilisation Kit (Invitrogen), according to the manufacturer’s instructions. Cells were incubated with anti-OCT3/4-Isoform A-PE antibody (Miltenyi) or isotype control IgG-PE (REA-PE, Miltenyi) and analysed using an LSRII analyser with Diva 8.02 software (BD). Cultures were used for injection only if >90% of the tested population expressed OCT3/4. Detailed information on antibodies used is presented in Table S1.

### 4.3. Animals and Teratoma Assay

Mice used in this study were 18–21-week-old NOD, Cg-*Prkdc^scid^ Il2rg^tm1Wjl^*/SzJ (NSG) males, 22-week-old NSG females, and 25-week-old NSG females engrafted with human CD34+ hematopoietic stem cells (HIS mice). All animals were purchased from the Jackson Lab (USA) and, upon arrival, were housed in sterile conditions in individually ventilated cages. All experiments were performed at Leiden University Medical Centre (LUMC) and were approved by the Dutch Central Commission for Animal experimentation (permit: ADV116002016735) and performed according to ARRIVE guidelines [36]. Group allocation is presented in Figure S1. Briefly, 1 × 10^6^ cells in 200 µL Matrigel mixed with cold mTeSR-E8 1:1 (Corning/Gibco) for hiPSCs and DMEM-F12 for 2102Ep were subcutaneously injected into the mouse’s right flank. This time point is referred to as T0. Animals receiving LU07+dox cells were given 2 mg/mL doxycycline (Sigma-Aldrich), with 10 mg/mL sucrose (Sigma-Aldrich) in drinking water to maintain construct expression, while animals receiving LU07 cells were provided with only sucrose in drinking water [18]. Water with additives was changed every other day. 

### 4.4. Plasma Sample Collection for mi-RNA Analysis

For each animal, plasma collection with tail bleeding was performed before cell injection (T-1) one week after injection, followed by bi-weekly collection throughout the entire experiment. Additional sample collection was performed at tumour removal if the previous collection was more than 7 days earlier. Blood was collected to heparin-containing collection tubes and centrifuged at 5000 rpm for 5 min; plasma was removed and stored at −80 °C until miRNA isolation and analysis. 

### 4.5. Tumour Growth Monitoring

Tumour growth was monitored weekly by palpation and measured with a digital calliper. The tumour volume (V) was calculated according to the formulation V = (W^2*L)/2 [37], where W is width, and L is length. 

Once the tumour reached ~2 cm^3^ (T1), it was surgically removed under anaesthesia and perioperative analgesia. T1 tumours were processed for histological analysis. The animal was kept alive up to 8 weeks after T1 removal and closely monitored for tumour growth and animal welfare. At the experimental endpoint (T2), the animals were sacrificed, and internal organs—and, if present, the tumour (T2)—were collected and, after fixation, embedded in paraffin for analysis. 

### 4.6. Tumour Histology

Tumour tissue was fixed in 4%PFA (Sigma-Aldrich) overnight at room temperature (RT) and embedded in paraffin. Paraffin sections (5µm thick) were prepared according to standard protocols and stained with haematoxylin and eosin (H&E) for morphological analysis, as described [18]. Evaluation based on H&E stain was performed by a European board-certified veterinary pathologist (D.C.F.S) and by one of the authors (M.B.). For immunohistochemical staining with various antibodies (Table S1), antigen retrieval was performed by heating the slides at 95 °C for 12 min either in citrate buffer pH 6.0 or Tris-EDTA buffer pH 9.0. Sections were permeabilised with 0.05%Tween or 0.1%Triton, blocked in 0.05%Tween–1%BSA for 1 h at RT, and incubated with primary antibodies in a blocking solution overnight at RT. Appropriate biotinylated secondary antibodies were diluted in a blocking solution and incubated for 1.5 h at RT. Signal amplification was performed by incubation with ABC-HRP Kit (Vector Laboratories) reagent, followed by DAB detection (Vector Laboratories) or BrightDAB (ImmunoLogic). Nuclei were counterstained with haematoxylin. A list of used antibodies is provided (Table S1). 

### 4.7. RNA Isolation and miRNA Analysis

miRNA from plasma samples and tumours was isolated as described [13]. For additional analysis of miRNAs, tumour sections were deparaffinised and dehydrated following standard procedures. Tissue was scraped off the glass slides and collected in TRIzol™ reagent (Invitrogen). Total RNA and miRNAs were retrieved using a Direct-zol™ RNA MicroPrep Kit (Zymo Research), according to the manufacturer’s protocol. miRNA levels were normalised using the endogenous control RNU48 [38]. 

### 4.8. Imaging and Quantification

Images were taken with Panoramic Scanner 250 (3DHISTECH) and CaseViewer 2.4 software. Brightness/contrast adjustments were made with Fiji software [39]. Figures and illustrations were assembled in Adobe Illustrator CS5.

### 4.9. Volume Calculation from Sections

To calculate tissue volume from sectioned material, we used all available serial sections (sectioned 1:10) stained with H&E of each tumour. Tissue area was measured on every slide using the ‘measure particles’ tool in Fiji software, multiplied by the thickness of the section (0.005 mm) and the distance between sections (0.05 mm). Since tumours varied in size and the number of sections obtained, the summarised values for each tumour were divided by the number of sections used for counting. The amount of sections used for each tumour was T182 *n* = 23; T183 *n* = 17; T184 *n* = 19; T185 *n* = 13; T186 *n* = 7; T187 *n* = 21; T188 *n* = 12; T189 *n* = 20; T190 *n* = 17; T191 *n* = 19; T192 *n* = 10; T193 *n* = 11; T194 *n* = 13; T195 *n* = 16; T196 *n* = 18; T197 *n* = 12; T198 *n* = 20; T199 *n* = 11.

### 4.10. Macrophage Counting

Selected slides from LU07, LU07+dox, and 2102Ep generated tumours were stained with macrophage marker F4-80 using mouse lung and spleen as positive controls. Positive cells were manually counted on the entire slide in the tumour parenchyma using CaseViewer software. The area of the section (in mm^2^) was calculated in Fiji using the ‘analyse particles’ option after threshold adjustment. Areas of the tumour at the edges which were not derived from hiPSCs (judged based on anti-human nucleus stain) were excluded from cell count and area measurement.

### 4.11. Statistical Analysis

All graphs and statistical analysis (2-way ANOVA test) were made in Prism 6.0c. Statistical analysis for macrophage counting was performed using the Mann–Whitney test.

## Data Availability

All data are available at request.

## Supplementary Materials

The following supporting information can be downloaded at: https://www.mdpi.com/article/10.3390/ijms23094680/s1.
