# Supplementary material for: Humanised Mice and Immunodeficient Mice (NSG) Are Equally Sensitive for Prediction of Stem Cell Malignancy in the Teratoma Assay
Source: Int J Mol Sci. 2022 Apr 23;23(9):4680. doi: 10.3390/ijms23094680 (PMC9105268; doi:10.3390/ijms23094680)
Supplement: Supplementary file 1 [file ijms-23-04680-s001.zip › manuscript.v6.supplementary.final version.pdf]

Supplementary Materials

Cell line	Cell type	Male (n)	Female (n)	
		NSG	NSG	HIS
2102Ep	hEC	T206-T208 (3)	T200-T202 (3)	T203-T205 (3)
LU07	hiPSC	T194-T196 (3)	T182-T184 (3)	T185-T187 (3)
LU07+dox	hiPSC	T197-T199 (3)	T188-T190 (3)	T191-T193 (3)

Figure S1. Overview of animal experimental groups based on the cell line injected in NSG or HIS mice. LU07 is a benign hiPSC cell line; LU07+dox is malignant due to presence of embryonal carcinoma resulting from activation of reprogramming transgenes, and 2102Ep is representative of an embryonal carcinoma cell line. hiPSC, human-**i**nduced pluripotent **s**tem **c**ell; hEC, **h**uman **e**mbryonal **c**arcinoma. T numbers represent individual animals; numbers in brackets indicate the number of animals injected.

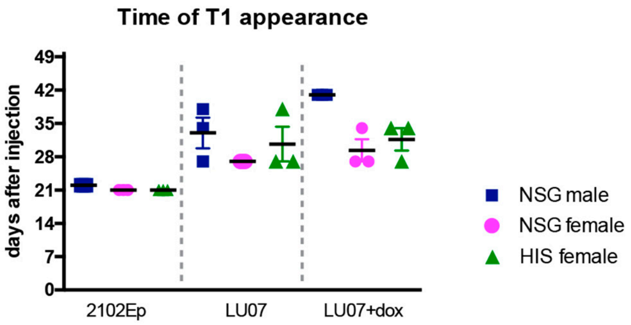

Figure S2. Time at which T1 tumours were first detectable. Day of the experiment in which the tumours were first detectable after injection of hPSCs (T0). Each point represents a single animal. Black bar horizontal bars indicate the mean; error bars indicate standard error of the mean.

A

Cell line	Male (n) NSG		Female (n) NSG		Female (n) HIS	
	T1	T2	T1	T2	T1	T2
2102Ep	3/3	2/3	3/3	3/3	3/3	3/3
LU07	3/3	0/3	3/3	0/3	3/3	0/3
LU07+dox	3/3	0/3	3/3	1*/3	3/3	1*/3

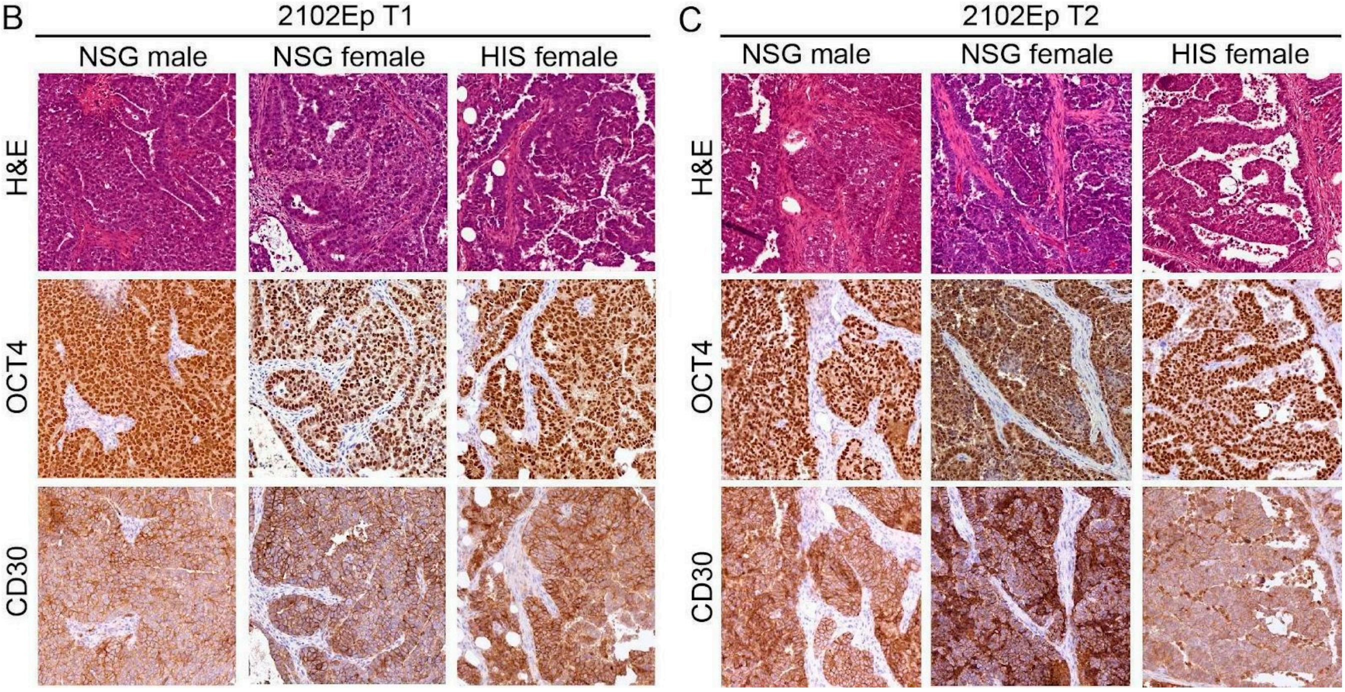

Figure S3. Analysis of tumours from 2102Ep injected animals:(**A**) an overview of the presence of T1 and T2 tumours in all animals; (**B**,**C**) representative pictures of H&E matched with OCT4- and CD30-stained sections of 2102Ep T1 and T2 tumours, respectively. *We could not verify that these were indeed tumours. They appeared as small fluid-filled cysts.

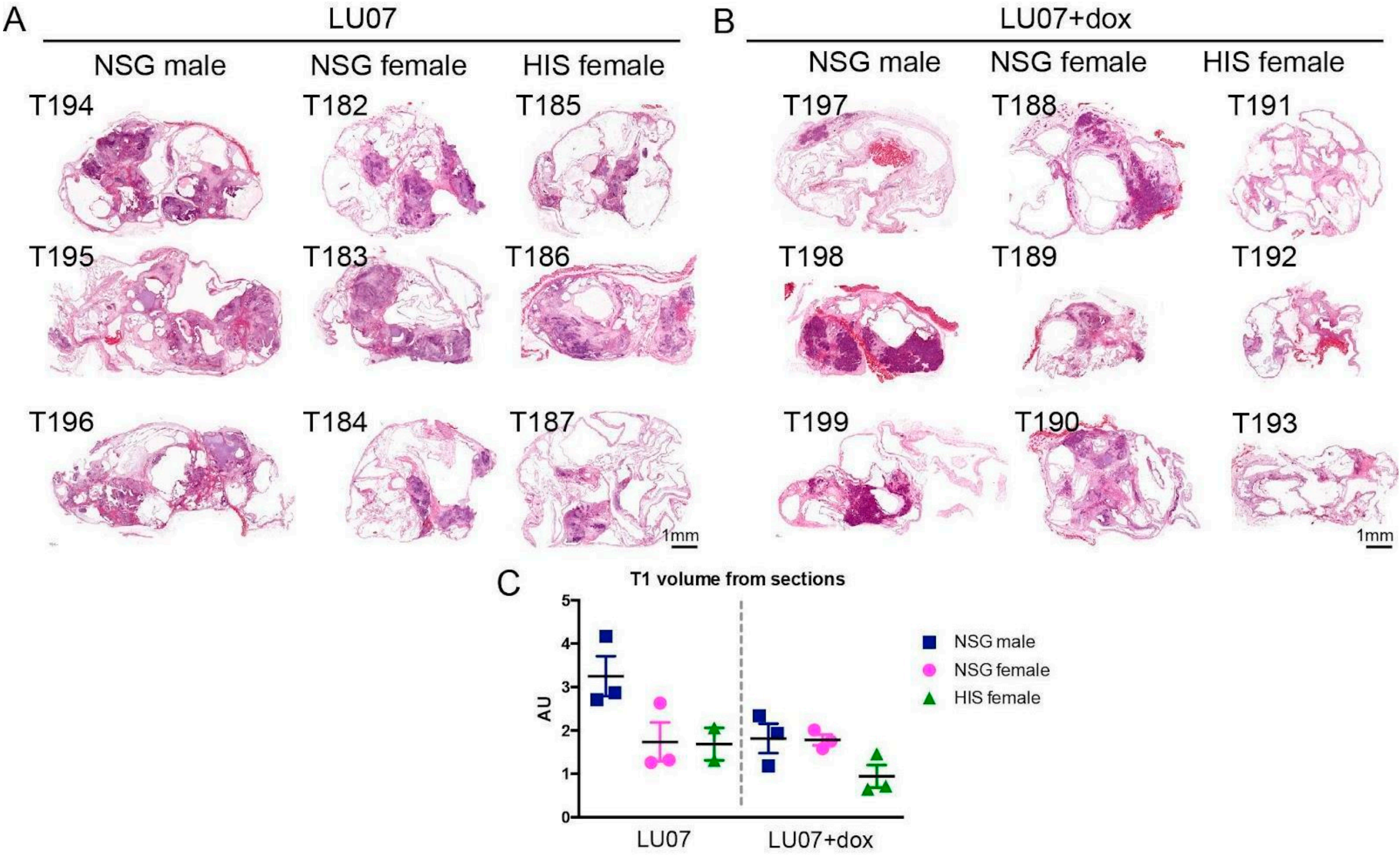

Figure S4. Tumour histology and volume measurement from sections of LU07 and LU07+dox tumours: (**A**,**B**) representative sections from tumours derived from LU07 and LU07+dox, respectively, showing overall tissue density; (**C**) quantification of the tissue volume calculated from all H&E-stained serial sections available for LU07 and LU07+dox tumours. Each data point represents one tumour. Note that there are no significant differences between animal groups within one cell line. Error bars indicate standard error of the mean.

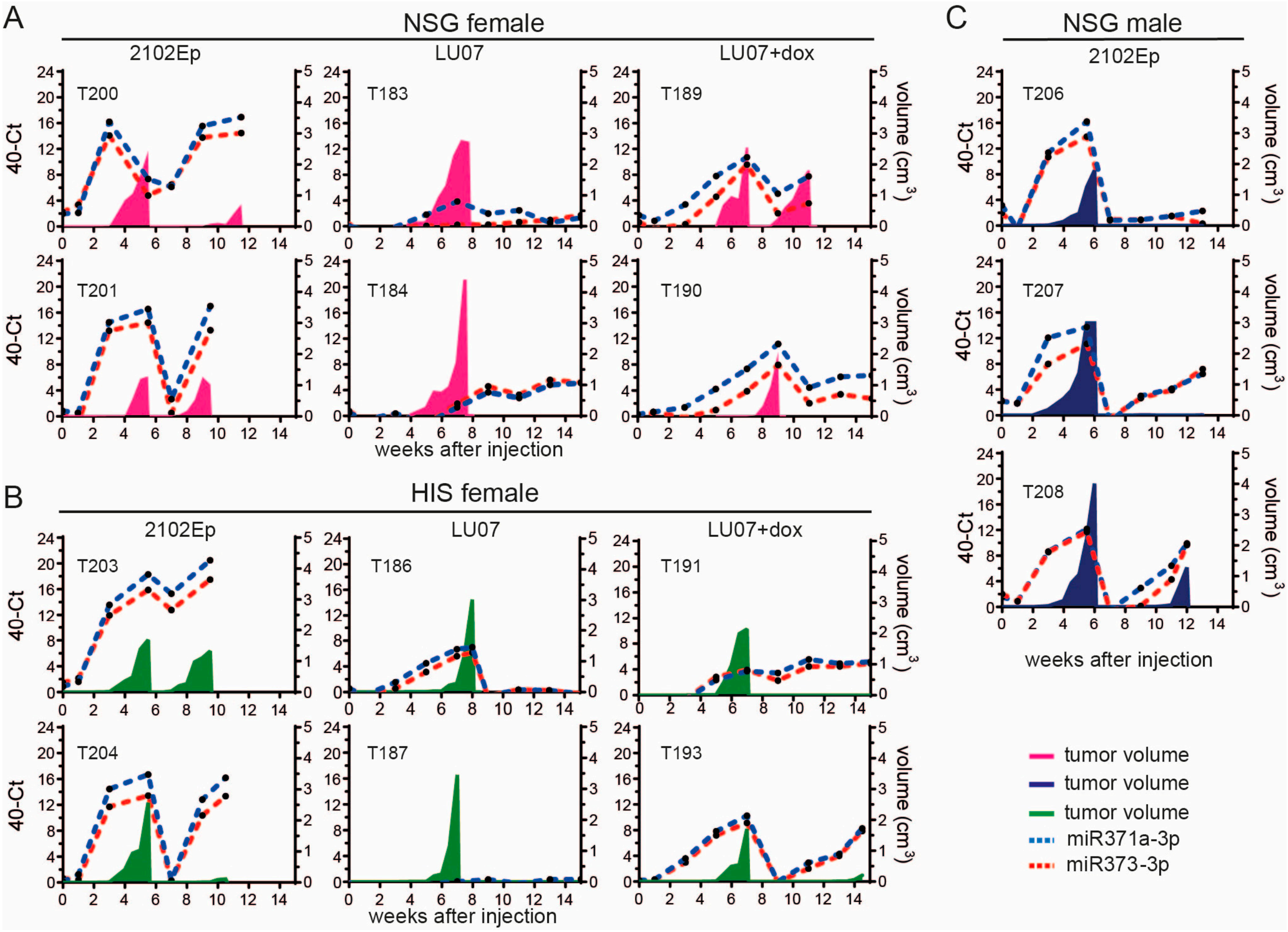

Figure S5. Time course of miRNA-371a-3p and miRNA-373-3p expression in the mouse plasma samples of the remaining two mice injected with 2102Ep, LU07, and LU07+dox cells: (**A**) relative levels (40-CT) of circulating miRNA-371a-3p and miRNA-373-3p in the plasma of NSG (**A**) and HIS females (**B**), and NSG males (**C**) xenografted with 2102Ep, LU07, and LU07+dox cells. Plasma samples were collected once every two weeks until the end of the experiment (max week 15). Tumour size is depicted in volume (cm^3).

Table S1. List of antibodies used in the study.

	Dilution	Manufacturer	Cat nr	Used For	Antigen Retrieval
**Primary antibodies**					
Anti OCT3/4-Isoform A-PE	1:50	Miltenyi	130-123-771	FACS	n/a
Mouse IgG1-PE	1:50	Miltenyi	130-117-098	FACS	n/a
Mouse anti-OCT4	1:100	Santa Cruz	sc-5279	IHC	Citrate pH = 6 l Tris-EDTA pH = 9
Goat anti-OCT4	1:100	Santa Cruz	sc-8628	IHC	Citrate pH = 6
Mouse anti-CD30	1:100	SantaCruz	sc-19658	IHC	Tris/EDTA pH = 9
Mouse anti-Human Nucleus	1:50	Abcam	ab190710	IHC	Citrate pH = 6 l Tris-EDTA pH = 9
Mouse anti-F4-80	1:200	Thermofisher	PA5-32399	IHC	Citrate pH = 6
**Secondary antibodies**					
Horse anti-mouse~biotin	1:200	Vector Labs	BA-2000	IHC	n/a
Horse anti-goat~biotin	1:200	Vector Labs	BA-9500	IHC	n/a
ABCkit~HRP	1:100	Brunschwig	PK-6100	IHC	n/a
