# Supplementary figures and images for: Humanised Mice and Immunodeficient Mice (NSG) Are Equally Sensitive for Prediction of Stem Cell Malignancy in the Teratoma Assay
Source: Int J Mol Sci. 2022 Apr 23;23(9):4680. doi: 10.3390/ijms23094680 (PMC9105268; doi:10.3390/ijms23094680)

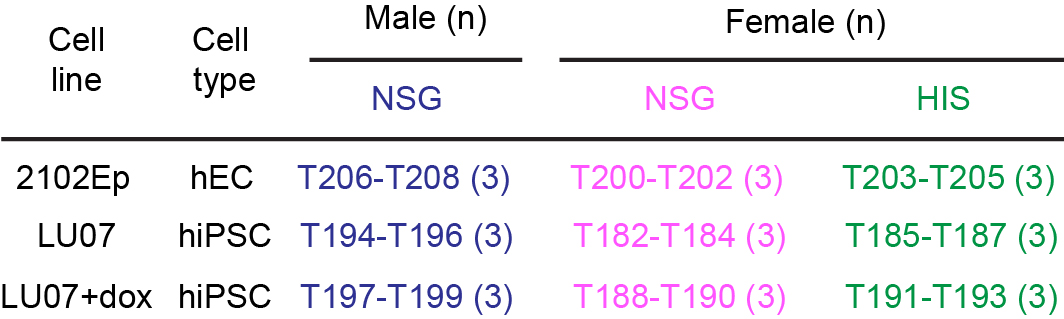

Supplement: Supplementary file 1 [file ijms-23-04680-s001.zip › Figure S1.jpg]

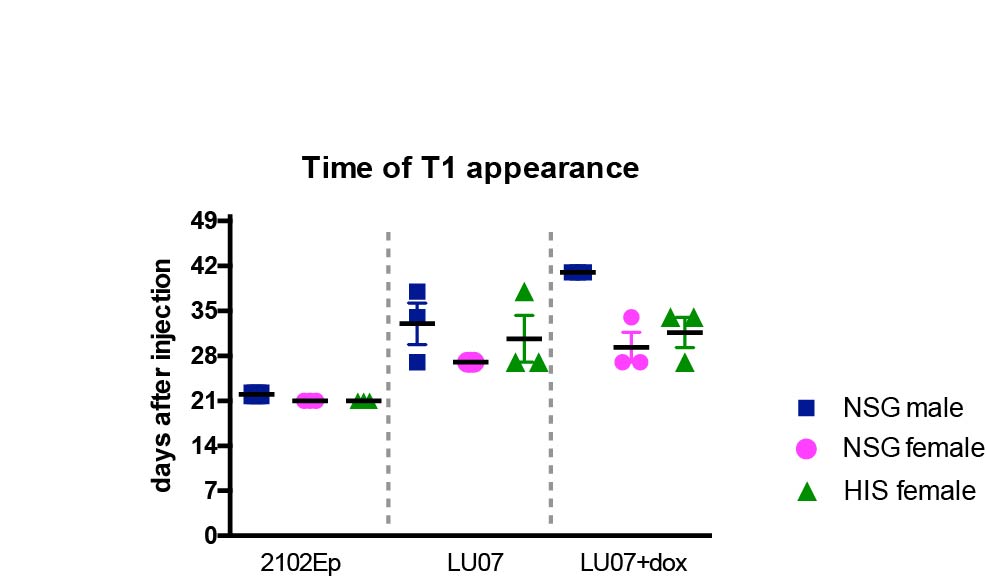

Supplement: Supplementary file 1 [file ijms-23-04680-s001.zip › Figure S2.jpg]

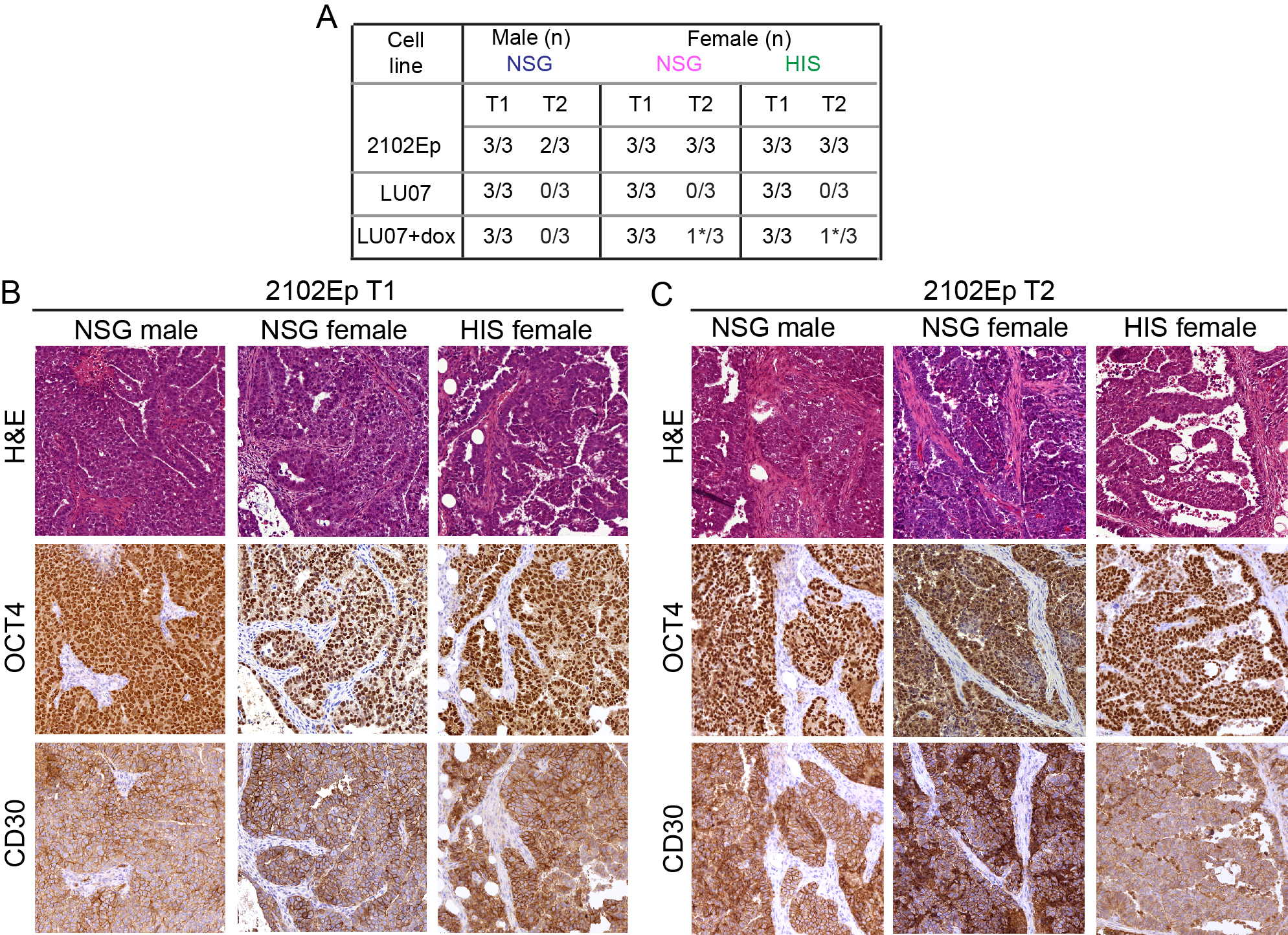

Supplement: Supplementary file 1 [file ijms-23-04680-s001.zip › Figure S3.jpg]

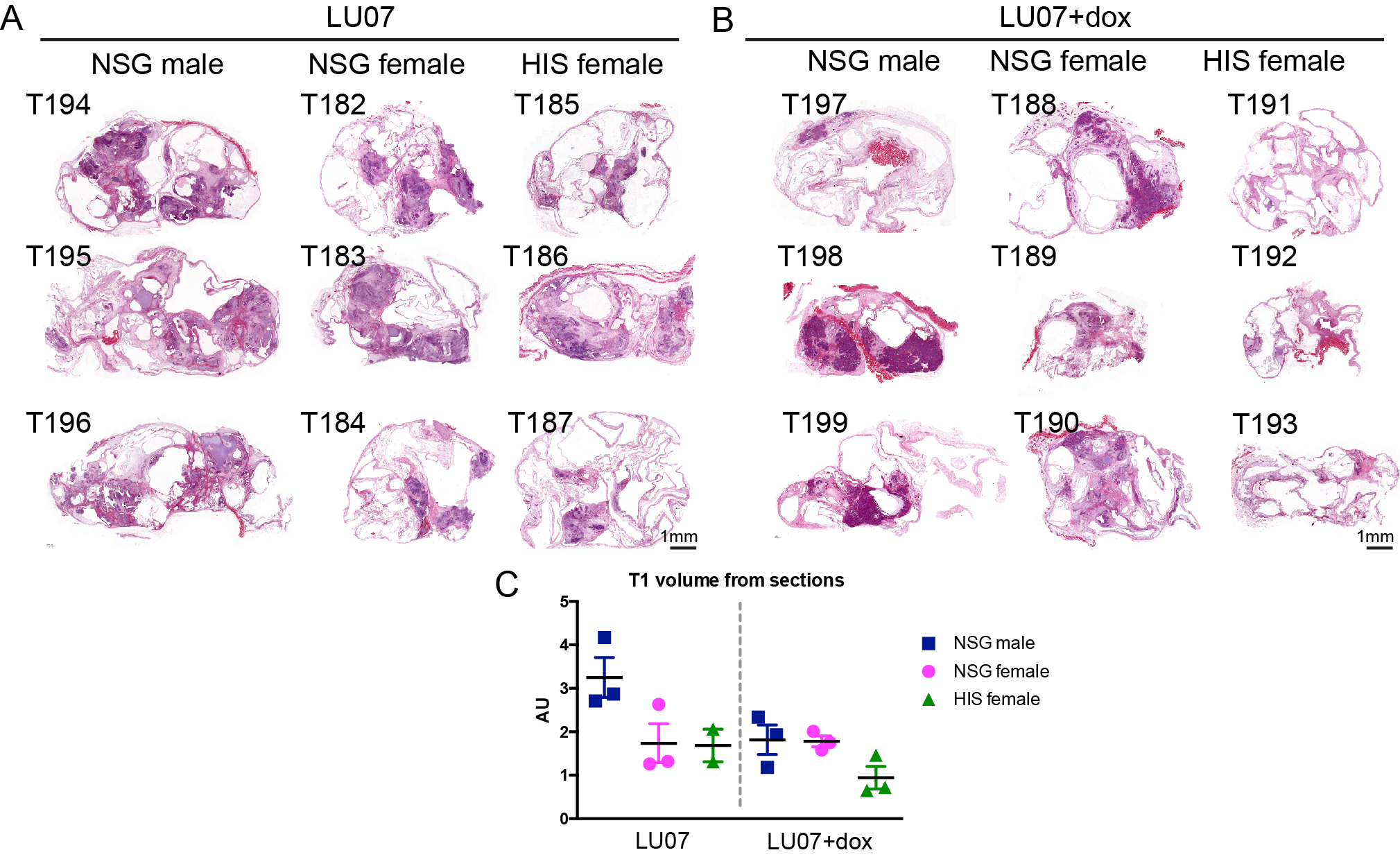

Supplement: Supplementary file 1 [file ijms-23-04680-s001.zip › Figure S4.jpg]

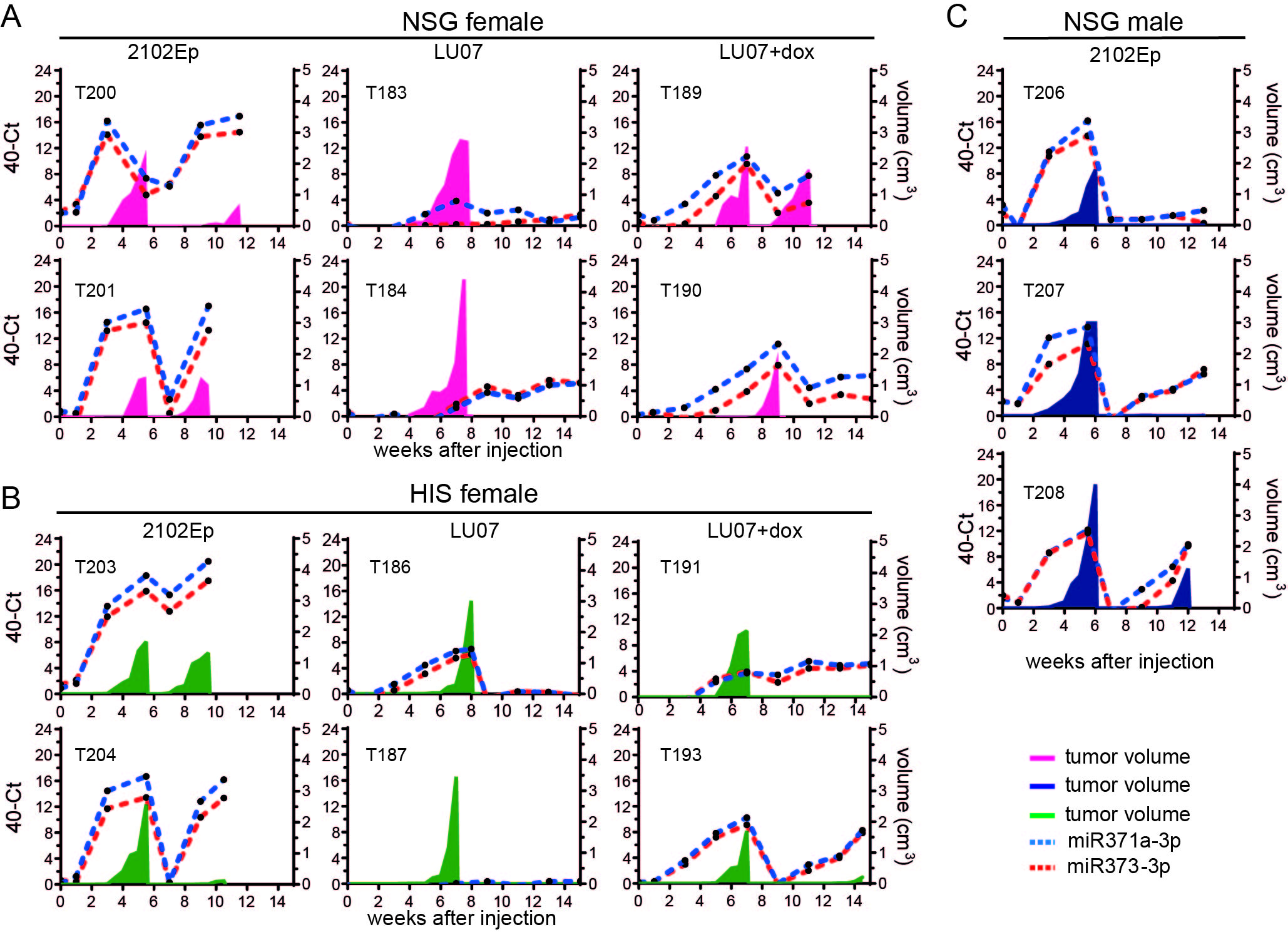

Supplement: Supplementary file 1 [file ijms-23-04680-s001.zip › Figure S5v2.jpg]
